# Supra- and Subgingival Microbiome in Gingivitis and Impact of Biofilm Control: A Comprehensive Review

**DOI:** 10.3390/antibiotics13060571

**Published:** 2024-06-20

**Authors:** Margarita Iniesta, Viviane Vasconcelos, Mariano Sanz, David Herrera

**Affiliations:** 1 ETEP (Etiology and Therapy of Periodontal and Peri-Implant Diseases) Research Group, Faculty of Dentistry, Complutense University of Madrid, 28040 Madrid, Spain; marsan@ucm.es (M.S.); davidher@ucm.es (D.H.); 2 Section of Graduate Periodontology, Department of Dental Clinic Specialties, Faculty of Dentistry, Complutense University of Madrid, 28040 Madrid, Spain; vivivasc@ucm.es

**Keywords:** microbiome, biofilm control, gingivitis, toothpaste, mouth rinse, metabarcoding

## Abstract

This comprehensive review aimed (1) to characterize the sub- and supragingival microbiome in patients with biofilm-induced gingivitis (including experimental gingivitis), (2) to assess its stability and evolution over time, and (3) to assess the impact of biofilm control measures on this stability. An electronic search of the MEDLINE^®^/PubMed^®^ database until December 2023 was conducted. NCBI Taxonomy, eHOMD 16S rRNA Reference Sequence, and Tree Version 15.23 databases were used to standardize taxonomic nomenclature. Out of 89 papers initially retrieved, 14 studies were finally included: 11 using experimental gingivitis as a model and three randomized clinical trials evaluating the impact of biofilm control measures. Among them, five characterized the subgingival microbiome, nine the supragingival microbiome, and one both the sub- and supragingival microbiome. In addition, five studies evaluated the effect of toothpaste, and four studies evaluated the effect of mouth rinses. The diversity and structure of the microbiome differed significantly between patients with periodontal health and those with biofilm-induced gingivitis (including experimental gingivitis). Those differences were not reversed through conventional oral hygiene measures. Specific antiseptic agents, especially if delivered as mouth rinses, may have an impact on the supra- and subgingival microbiome in gingivitis.

## 1. Introduction

Oral health is a crucial component of an individual’s overall well-being; therefore, the maintenance of a balanced oral microbiome becomes important for overall health sustainment. In fact, disturbance of the oral microbial balance may lead to a variety of local and systemic conditions [1], such as caries and periodontal diseases, which are among the most prevalent conditions affecting human beings. Among periodontal diseases, biofilm-induced gingivitis stands as the most common oral disease, affecting nearly 95% of the population [2]. If left untreated, biofilm-induced chronic gingivitis may progress to periodontitis, a condition characterized by non-resolving inflammation leading to irreversible attachment loss and alveolar bone destruction, which may result in tooth loss [3].

Biofilm-induced gingivitis is caused by a dysbiosis of the supra/subgingival microbiota, i.e., a change in the relative abundance of individual microbiota species compared with their abundance in periodontal health. This dysbiosis leads to alterations in the host–microbiota interaction, which is sufficient to initiate this disease [4]. In biofilm-induced gingivitis, an increase in the amounts of certain subgingival microorganisms is observed, resulting in a change in the composition of the overall subgingival microbial community. These changes in composition create an environment conducive to the overgrowth of more pathogenic species, leading to inflammation of the gingival tissues [5].

As biofilm-induced gingivitis develops, the microbial composition of the subgingival biofilm shifts from a population dominated by gram-positive *Streptococcus* spp. to one where gram-negative anaerobes become predominant. These include species of *Capnocytophaga*, *Selenomonas*, *Veillonella*, *Campylobacter*, *Fusobacterium*, and *Prevotella*, among others [6]. The inflammatory exudate released to the gingival sulcus further favors the growth of other gram-negative bacteria since they use the products resulting from the inflammatory process as a source of nutrients [7]. In this feedback loop, the inflammatory process induced by the changes in the composition of the dental biofilm will perpetuate both the inflammatory process and further changes in the biofilm composition.

Therefore, the removal and/or biofilm control is the main element in the prevention and treatment of biofilm-induced gingivitis [3], which also becomes the most relevant approach to primarily prevent periodontitis. Different oral hygiene measures, including antiseptic agents, have demonstrated significant effects on both clinical inflammation [8,9] and the oral microbiome [10]. The latter review evaluated the effect of antiseptics on the oral microbiome in the general population without distinguishing between subjects with gingivitis or periodontitis. Consequently, the mechanisms by which different biofilm control measures affect the supra/subgingival microbiome in patients with biofilm-induced gingivitis remain elusive.

On the other hand, reviews that focused on the oral microbiome and included patients with gingivitis [11] did not analyze the temporality of the observed changes.

It was, therefore, the aim of the present comprehensive review (1) to characterize the sub- and supragingival microbiome in patients with biofilm-induced gingivitis (including experimental gingivitis), (2) to assess its stability and evolution over time, and (3) to assess the impact of biofilm control measures on this stability.

## 2. Results

### 2.1. Study Characteristics

**Figure 1:**
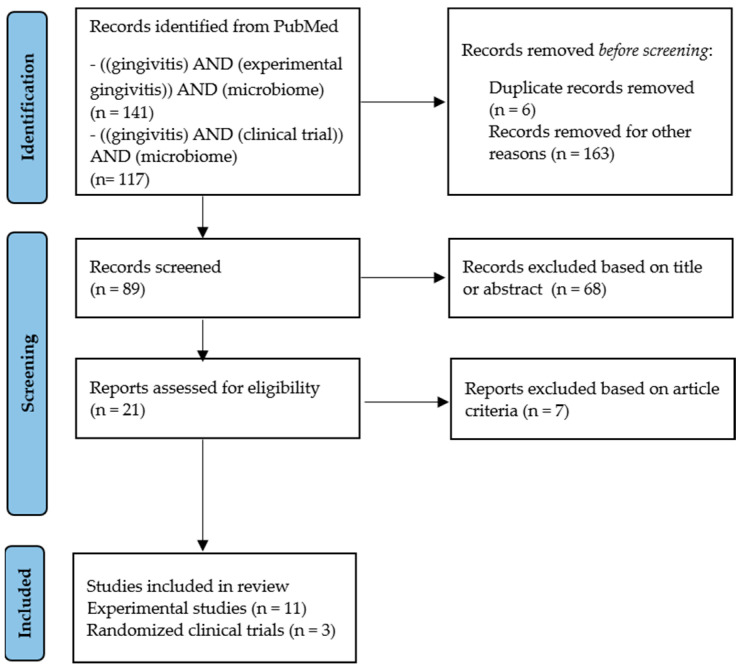
Number of articles found in the database, descriptors, criteria, and sample selected for comprehensive review.

A total of 89 papers were initially identified, and 14 studies were finally selected. From this selection, 11 studies used the experimental gingivitis model, and three were randomized clinical trials (RCTs) (Figure 1).

The experimental studies had different designs (Table S1). Four studies were designed with pre-induction, gingivitis induction, and gingivitis resolution phases [12,13,14,15]; four studies had only pre-induction and gingivitis induction phases [16,17,18,19]; two studies had gingivitis induction and gingivitis resolution phases [20,21]; and one study presented only the phase of gingivitis induction [22].

The studies evaluating antiseptic agents were designed as RCTs (*n* = 3) or experimental studies (*n* = 5, mentioned above). Studies evaluating or having subject groups using specific toothpaste were (a) two RCTs evaluating sodium fluoride [23,24] and (b) three experimental studies with toothpaste including sodium fluoride and stannous fluoride [15], sodium monofluorophosphate (MFP) [13], and triclosan/copolymer [12]. Studies evaluating mouth rinses were (a) two experimental studies, one assessing N-acetyl cysteine (NAC) and chlorhexidine (CHX) [21] and another with cetylpyridinium chloride (CPC) [17], and (b) two RCTs, one with CPC plus essential oils [25] and another with CPC [23]. Table S2 depicts the characteristics and design of the RCTs.

Relevant methodological characteristics are shown in Table S3.

### 2.2. Microbiome Changes in Experimental Gingivitis

#### 2.2.1. Alpha Diversity

In the subgingival microbiome, the number of observed operational taxonomic units (OTUs)/amplicon sequence variants (ASVs) increased from day 0 (cessation of oral hygiene) to 21 days, when gingivitis was well established, with no statistically significant differences [13,14,21]. Regarding the dynamics of colonization, a statistically significant increase in the number of species was observed at 14 days compared with baseline (both the number of observed ASVs and the Chao1 index); however, this increase was not significant at 21 days [13]. Conversely, Schincaglia et al., (2017) reported a statistically significant increase in the number of observed OTUs at 21 days [12].

Regarding abundance and evenness, the Shannon index increased from the day of oral hygiene cessation to the end of the gingivitis induction phase (i.e., from day 0 to day 21) [14,15,21]. This increase at day 21 was statistically significant compared with baseline in two studies [12,13].

When subjects were grouped according to gingival index severity at the end of the 21-day period, the Shannon index showed statistically significant differences between subjects with high and medium scores compared with the low-scoring group [18].

Using Faith’s phylogenetic diversity index, a significant increase was observed in the subgingival samples on days 14 and 21 compared with day 0 [13].

In the supragingival microbiome, no statistically significant increase in the number of observed ASVs was observed from day 0 to day 21. The magnitude of the change was greater in the subgingival than in the supragingival samples, although not significant in either case [14]. However, an OTU-based study reported a statistically significant increase in genus richness from day 0 to day 21 [17]. Using the Shannon index, the increase from day 0 to day 21 was not significant in supragingival samples [14]. Conversely, Teng et al., (2016) reported a significant increase in the Shannon diversity of supragingival samples from day 0 to day 21 [17]. Kistler et al., (2013) also observed that the Simpson’s inverse index was significantly higher on day 14 (at the end of the induction phase) compared with day 0 and day 7, but no significant differences were reported between day 0 and day 7 [22].

#### 2.2.2. Beta Diversity

In subgingival samples, beta diversity showed changes in community composition over the 21-day period. Based exclusively on sequence distances (unweighted UniFrac distance), compositional differences between periodontal health and gingivitis could already be detected as early as 4 days and were maintained through 21 days [13]. Regarding abundance, using the abundance–Jaccard distance as a metric, compositional differences were reported between day 0 and the end of the induction phase [21]. Furthermore, a significantly different clustering (Bray–Curtis dissimilarity) was also observed when comparing samples with the highest and lowest gingival index scores [18].

When assessing the compositional difference in the supragingival biofilm using Principal Components Analysis (PCA), there were changes from day 4 of the gingivitis induction phase, and these were maintained until the end of the experiment [20]. Using the genus-level Bray–Curtis dissimilarity, this shift in the supragingival microbiome was observed as early as day 1 of the gingivitis induction phase [19]. Other studies also corroborated changes in the microbial community between day 0 and day 21 using thetaYC and weighted UniFrac metrics [16,22], thetaYC [13] and PCA [17].

In terms of variability among different oral sites, intrasubject dissimilarities (Bray–Curtis dissimilarities) were consistently lower than between-subject dissimilarities at any temporal point [14].

#### 2.2.3. Phyla

In the subgingival samples, bacterial phyla with a relative abundance of ≥1% at the beginning of the induction phase were *Firmicutes*, *Bacteroidota*, *Proteobacteria*, *Fusobacteriota*, *Actinobacteriota*, and *Spirochaetota* [14]. Other phyla < 1% were *Candidatus Saccharibacteria* (formerly known as TM7) [21], *Thermodesulfobacteriota* (formerly *Desulfobacteriota*), *Cyanobacteriota*, *Campylobacterota*, and *Patescibacteria* group [14]. The relative abundance of *Firmicutes*, *Actinobacteriota*, *Proteobacteria*, *Thermodesulfobacteriota*, and *Cyanobacteriota* decreased during the 21 days [13,14]. However, Al-Kamel et al. (2019) observed an increase in the abundance of the phylum *Proteobacteria*. In contrast, *Bacteroidota*, *Fusobacteriota*, *Saccharibacteria*, *Spirochaetota*, *Campylobacterota*, and *Patescibacteria* group increased in their relative abundance [13,14,21].

The dynamics of these changes may vary depending on the phyla. Bamashmous et al., (2021) reported that *Firmicutes* reached the lowest abundance at 14 days and *Actinobacteriota* at 7 days. However, *Bacteroidota* reached a peak at 14 days and *Fusobacteriota* at 21 days [13]. In the study by Hall et al., (2023), *Firmicutes*, *Actinobacteriota*, and *Proteobacteria* reached the lowest amount at 21 days; *Fusobacteriota*, *Campylobacterota*, and *Patescibacteria* group reached a peak at 14 days and *Spirochaetota* at 21 days; and *Bacteroidota* reached a plateau between days 7 and 21 [14].

In supragingival samples, bacterial phyla with a relative abundance of ≥1% at the beginning of the induction phase were *Firmicutes*, *Proteobacteria*, *Bacteroidota*, *Actinobacteriota*, *Fusobacteriota*, and *Spirochaetota* [14]. The relative abundance of *Proteobacteria*, *Actinobacteriota*, *Spirochaetota*, *Thermodesulfobacteriota*, and *Cyanobacteriota* decreased significantly during the 21-day period, and the relative abundance of *Bacteroidota*, *Fusobacteriota*, *Campylobacterota*, *Patescibacteria*, and *Saccharibacteria* increased [14,16,22]. Although Huang et al., (2014) observed a higher abundance of the phylum *Spirochaetota* at day 21, the change was not significant [16]. *Firmicutes* remained stable with few changes at day 21 [14]. However, in the study by Huang et al., (2014), a statistically significant reduction in this phylum occurred during the induction phase [16].

#### 2.2.4. Genera and Species

##### *Bacteroidota* 

One of the phyla with the highest relative abundance during gingivitis induction was *Bacteroidota*. Its abundance was due to a significant increase in the genera *Prevotella*, *Porphyromonas*, *Alloprevotella*, and *Tannerella* at the subgingival [13,14,15,18,21] and supragingival levels [14,16,17]. In supragingival samples, *Capnocytophaga* was increased [20], and *Lautropia* was decreased [17]. Huang et al., (2021) and Belstrøm et al., (2018), however, did not observe an increase in the genus *Tannerella* and *Prevotella*, respectively, in the supragingival biofilm [16,20]. The genus *Prevotella* was one of the most abundant genera at the end of gingivitis induction in subgingival samples [14]. Table 1 depicts the changes in the different species reported in the different studies.

##### *Fusobacteriota* 

An increase in the genera *Fusobacterium* and *Leptotrichia* was observed at subgingival [14,18,21] and supragingival levels [14,16,17,19]. In subgingival samples, *Fusobacterium* was also one of the most abundant genera at the end of gingivitis induction at these sites [14]. This increase was mainly due to the increase in the relative abundance of the species listed in Table 1.

##### *Spirochaetota* 

The phylum *Spirochaetota* increased its relative abundance by increasing the genus *Treponema* at the subgingival level [13,14]. In supragingival sites, *Treponema* was found to increase significantly in one study [16] and decrease significantly in another [14]. The dynamics of various *Treponema* species are shown in Table 1.

##### *Patescibacteria* Group

This group represents a major bacterial phylogenetic group that includes various uncultivated lineages, including 35 phyla [26]. In this clade, an increase in the genus *Gracilibacteria* (*Candidatus Gracilibacteria* phyl., formerly known as GN02) was found in the sub- and supragingival areas [14]. The genus *Saccharibacteria* (*Candidatus Saccharibacteria* phyl., formerly known as TM7) was increased in subgingival [13] and supragingival biofilms [16,19] (Table 1).

##### *Firmicutes* 

Within the reduction of the phylum *Firmicutes*, two different dynamics occurred. In general, the genera of the class *Bacilli* decreased, and the class *Clostridia* and *Negativicutes* increased in both the sub- and the supragingival biofilms. However, the studies showed minor differences in their results (Table 2). The changes occurring at the species level are shown in Table 3.

##### *Proteobacteria* 

The genera *Neisseria*, *Haemophilus*, and *Lautropia* decreased, and *Aggregatibacter* increased, in both the sub- [13,14,18,21] and the supragingival areas [14,16,17,19,20]. Table 3 shows the changes in species.

##### *Actinobacteriota* 

The phylum *Actinobacteriota* decreased, while the genera *Actinomyces* and *Rothia* reduced in the subgingival [12,13,14,21] and the supragingival biofilms [14,16,17,19,20]. In supragingival sites, the genus *Corynebacterium* [20] increased. Table 3 shows the changes at the species level.

### 2.3. Impact of Oral Hygiene Products

#### 2.3.1. Toothpastes

Three of the experimental gingivitis studies evaluated the effect of toothpaste on the subgingival microbiome at 14 days (after PMPR) [13] or 21 days [12,15] after the completion of the induction phase. Two RCTs evaluated the impact of toothpaste on supragingival samples [23,24].

After 21 days of brushing with triclosan/copolymer [12] or stannous fluoride toothpaste [15], alpha diversity showed either a significant [12] or non-significant decrease [15] compared with the end of the induction phase. In addition, the stannous fluoride toothpaste showed lower alpha diversity than the control group (sodium fluoride toothpaste) [15]. Statistically significant reductions were observed for certain species with the stannous fluoride toothpaste compared with the control group. The phylum *Bacteroidota* and *Spirochaetota* were significantly lower in the stannous fluoride group compared with the control group. A similar trend was observed at the genus level for *Treponema* and *Bacteroidales*_[G2] and at the species level for *Porphyromonas endodontalis* and *Tannerella forsythia* [15].

After PMPR and the use of an MFP paste, the trend was similar: a significant decrease in alpha diversity (number of observed ASVs, the Chao1, Shannon, Simpson’s inverse, and Faith’s phylogenetic diversity indices) and also in beta diversity (unweighted UniFrac distance matrices) was observed with respect to day 21 (end of experimental gingivitis). The trend in phyla was a decrease in *Bacteroidota* (genus *Tannerella*) and *Fusobacteriota* and an increase in *Actinobacteriota* (genera *Actinomyces* and *Rothia*) and *Firmicutes* (genera *Gemella*, *Veillonella*, and *Streptococcus* increased while *Selenomonas* decreased) [13].

One of the RCTs [23], evaluating supragingival samples, included one arm (control group) only using a sodium fluoride toothpaste, and the Shannon index of the supragingival biofilm remained largely stable over a 27-day period. Moreover, different supragingival microbial community profiles could not be observed using the Jensen–Shannon divergence distance. Nevertheless, the genera *Leptotrichia*, *Actinobaculum*, and *Saccharibacteria* decreased significantly, while the genus *Actinomyces* showed a statistically significant increase at the end of the 27-day study period [23].

In the second RCT [24], control subjects brushed their teeth with a toothpaste containing sodium fluoride and zinc chloride during the 28-day clinical trial. A very limited impact on plaque microbiota was observed. Minor non-significant changes were observed in the abundance of *Actinobacteriota*, with an increase in the genera *Actinomyces* and *Corynebacterium*, and a reduction in the abundance of *Firmicutes*, *Fusobacteriota* (genera *Leptotrichia* and *Fusobacterium*), and *Bacteroidota* (genus *Capnocytophaga*).

#### 2.3.2. Mouth Rinses

Two experimental gingivitis studies evaluated the effect of mouth rinses: 1.25% NAC versus 0.20% CHX [21] in subgingival samples and 0.07% CPC [17] in supragingival samples. Two RCTs evaluated the impact of mouth rinses: 0.07% CPC [23] and CPC plus essential oils [25], both in supragingival samples.

In an experimental gingivitis study, the subjects were randomly assigned to use NAC or CHX mouth rinses at the end of the induction phase [21]. Alfa diversity, as assessed by the number of observed OTUs, Chao1, and Shannon indices after 14 days, was associated with a significant decrease in the CHX group, while the use of NAC did not produce a significant change. Based on the Jaccard distance matrix (beta diversity), the day 14 samples from the NAC group showed the same compositional pattern as the gingivitis-associated samples on day 21 (end of the gingivitis induction phase), while the CHX samples formed a separate group. Regarding the temporal change of the subgingival microbiome, some slight taxonomic changes were observed in the NAC group but without statistically significant differences. In contrast, in the CHX group, there was a significant decrease in the abundance of the phyla *Candidatus Saccharibacteria* (by decreasing the genera *Saccharibacteria*_(TM7)_[G-3] and *Saccharibacteria*_(TM7)_[G-1])*, Candidatus Absconditabacteria* (by reducing the genus *Absconditabacteria*_(SR1)_[G-1]), and *Actinobacteriota* (by reducing the genera *Corynebacterium*, *Actinomyces*, and *Propionibacterium*). At the genus level, this group was also associated with a significant increase in the relative abundance of *Capnocytophaga* and a decrease in the relative abundance of *Stomatobaculum*, *Selenomonas*, *Lachnospiraceae*_[G-3], *Lachnoanaerobaculum*, *Gemella*, *Veillonella*, *Tannerella*, and *Cardiobacterium.* Changes at the species level are shown in Table 4.

In another experimental gingivitis study, but with supragingival samples [17], subjects rinsed with CPC (test group) or water (control group) without any other oral hygiene practices during the gingivitis induction phase. They observed that the alpha diversity (genus richness and Shannon index) of the CPC group remained stable over the 21 days of the study. However, this CPC group showed a statistically significant lower beta diversity than the control group. Several genera were significantly inhibited, including *Porphyromonas*, *Peptostreptococcus*, *Prevotella*, *Peptococcus*, *Selenomonas*, *Solobacterium*, *Absconditabacteria*_(SR1)_[G-1], *Tannerella*, *Saccharibacteria*_(TM7), uncultured *Lachnospiraceae*, *Atopobium*, *Megasphaera*, *Mogibacterium*, *Moraxella*, *Gemella*, *Oribacterium*, and *Shuttleworthia*; and genera such as *Haemophilus*, *Lautropia*, *Neisseria*, *Capnocytophaga*, and *Propionibacterium* were significantly increased in subjects in the CPC group [17].

The two RCTs evaluated supragingival samples and assessed CPC without [23] and with essential oils [25]. Significant decreases in the alpha (Shannon index) and beta diversity (Jensen–Shannon divergence distance) of the samples were observed between day 11 and the end of the study with the use of a CPC mouth rinse [23]. The genera *Olsenella*, *Veillonellaceae*, *Leptotrichia*, *Actinomyces*, *Prevotella*, unclassified *Bacteroidaceae*, *Campylobacter*, *Actinobaculum*, *Peptococcus*, *uncultured Lachnospiraceae*, *Tannerella*, *Selenomonas*, and *Saccharibacteria*_(TM7) decreased significantly, while *Rothia*, *Lautropia*, and *Streptococcus* increased [23]. A CPC plus essential oils mouth rinse [25] was also tested after PMRP. At 12 weeks, alpha diversity as measured by the number of observed OTUs, Shannon index, and Faith’s phylogenetic diversity showed no statistically significant differences between groups, but beta diversity (weighted UniFrac distance) was significantly different in the CPC plus essential oils group. The species that were significantly reduced in this group were *Corynebacterium matruchotii*, *Corynebacterium durum*, various *Actinomyces*, *Fusobacterium*, *Leptotrichia*, *Capnocytophaga*, *Neisseria*, *Streptococcus*, *Aggregatibacter*, *Porphyromonas*, *Terrahaemophilus aromaticivorans* (now considered a synonym for *Haemophilus parainfluenzae* [28,29]), and *Lautropia* [25]. An overview of decreasing and increasing species after the use of CPC plus essential oils is shown in Table 4.

## 3. Discussion

Considering that there are estimated to be at least 774 oral bacterial species, of which 58% have been named, 16% are unnamed but cultivated, and 26% consist of uncultivated phylotypes [30], the present comprehensive review was designed to characterize the changes in the microbiome, analyzed by next-generation sequencing, that occur in biofilm-induced gingivitis (including experimental gingivitis), and how these changes may be influenced by normal oral hygiene practices. The collected information has shown that alpha and beta diversity in gingivitis is different and higher than in periodontal health and that these differences are more evident in the subgingival biofilm compared with the supragingival biofilm. The retrieved data present a dynamic of changes in the different phyla, genera, and species that are compatible with the concept of dysbiosis. In addition, various antiseptic products, especially if delivered as mouth rinses, can attenuate these changes, both in the subgingival and in the supragingival biofilms.

In the current era of microbiology, next-generation sequencing is a major breakthrough in periodontal research, expanding our understanding of the role of both cultured and uncultured bacteria. In contrast to targeted methods, such as PCR, checkerboard DNA hybridization, or microarrays, which do not permit the identification of previously undiscovered species, high-throughput sequencing technology has the capacity to detect the presence of virtually all microorganisms [31], thereby representing a truly non-targeted method. This makes high-throughput sequencing-based studies, such as metabarcoding and shotgun metagenomics, very suitable to provide the most comprehensive insight into the changes occurring in the supra- and subgingival microbiome of biofilm-induced gingivitis.

### 3.1. Higher Microbiome Diversity in Gingivitis Than in Periodontal Health

In subgingival samples, there is an increase in the relative abundance of certain species compared with periodontal health, which leads to a taxonomic shift at the phylum, genus, and species level. This increase in taxa would occur in a more balanced manner than that observed in periodontal health (where there is a dominance of the genus *Streptococcus* [14]), resulting in greater evenness and, therefore, greater diversity. There would be not only an increase in abundance but also a gain in species, such as *Selenomonas* spp., *Saccharibacteria*_(TM7)_[G-1] spp., *Mitsuokella* spp., *Oribacterium* spp., and *Dialister micraerophilus*, among others [21]. This species turnover between gingivitis and periodontal health may first be observed 1–4 days after the interruption of oral hygiene, but it would be significantly detectable 14 days after biofilm accumulation [13]. At the supragingival level, the same trend of increasing diversity was observed, but to a lesser extent.

Another interesting piece of information is that there was less variability in the composition of samples from the same patient than between samples from different patients. This could mean that the composition of the human oral microbiome is influenced by host genetic and/or environmental factors [32,33].

Once the primary colonizers are established in the biofilm, there is a change in the metabolome of the biofilm [19], which provides nutrients for bacterial growth, leading to further development of the biofilm. Within the first few days, the amount and variety of cytokines released into the environment is lower than in the following days, and the clinical status of the gingival tissue is compatible with periodontal health [19]. As the biofilm matures, it begins to induce a clear pro-inflammatory response [34], which leads to the clinically detectable inflammation of the gingival tissue [12]. Within biofilm interactions, commensal species, such as *Prevotella* and *Fusobacterium* spp., would begin to increase their relative abundance [6,14] and alter their gene expression [18,35], becoming virulent.

Therefore, periodontal health status represents an optimal opportunity to control biofilm accumulation, forming the best strategy for the primary prevention of gingivitis. Once gingivitis is established, preventive measures are also needed to prevent progression to periodontitis (primary prevention of periodontitis) or to prevent the recurrence of inflammation in cases of periodontitis (secondary prevention of periodontitis) [36]. The preventive approach should not only be based on the elimination of already formed supra- and subgingival biofilms (PMPR, applied both sub- and supragingivally), promotion of oral hygiene measures (oral hygiene instructions and motivation), and appropriate supportive periodontal care in cases of periodontitis but should also be based on the intervention of modifiable risk factors, such as smoking [3]. Smoking is also a risk factor at the microbiome level, as it facilitates the proliferation of disease-associated bacteria [37]. However, this effect on the oral microbial ecosystem could be reversed by the cessation of this habit [38].

### 3.2. Development of a More Complex Microbiome in Gingivitis

The dynamic shift that occurs during gingivitis seems to be based on an increase in some gram-negative phyla, such as *Bacteroidetes* and *Fusobacteria*, and a decrease in gram-positive phyla, such as *Firmicutes* and *Actinobacteriota*. Furthermore, changes in the microbiome reflected changes in gingival tissue inflammation [13,16,17,18], i.e., the greater the inflammation, the greater the alteration in the microbiome.

During the development of gingivitis, different bacterial species of the same genus tended to show identical patterns of change in their relative abundance, except for several species of the genera *Capnocytophaga*, *Treponema*, *Actinomyces*, *Neisseria*, and *Streptococcus*. Thus, species of the genera *Prevotella*, *Porphyromonas*, *Tannerella*, *Fusobacterium*, *Leptotrichia*, *Absconditabacteria*_(SR1)_[G-1], *Saccharibacteria*_(TM7)_[G-1], *Selenomonas*, *Clostridiales*, *Cardiobacterium*, and *Propionibacterium* increased, and species of the genera *Haemophilus*, *Corynebacterium*, *Kingella*, and *Rothia* decreased significantly at both the sub- and supragingival levels. In fact, the genus *Rothia* was negatively correlated with clinical inflammation [12,22]. The hypothesis would be that *Rothia* spp. could promote periodontal health via nitrate reduction by depleting the abundance of certain pathogens, such as *Porphyromonas gingivalis*, *Fusobacterium nucleatum*, and *Aggregatibacter actinomycetemcomtans* [39].

On the other hand, different species of the genus *Streptococcus* exhibited different dynamics. One of the species that increased in gingivitis was *Streptococcus cristatus*. This species has shown certain surface proteins that may interfere with the expression of the *FimA* gene of *P. gingivalis* [40,41], thereby disrupting the colonization of the biofilm by this bacterium and showing an antagonistic relationship between them [42]. This might explain why the abundance of *P. gingivalis* is not increased in gingivitis, and in contrast, an increase in *Porphyromonas catoniae* is detected. Another fact to consider is that species belonging to the genus *Streptococcus* have different responses to enterobactin from *Rothia mucilaginosa* [43].

### 3.3. Stability of the Gingivitis Microbiome

The oral microbiome composition, including the microbiome of gingivitis, is dynamic. It may be influenced by age [44], diet [45], hygiene habits, or even the dominant microbiota [16]. Experimental gingivitis studies confirmed that the increase in different taxa occurred at different rates, with some taxa taking longer to increase or decrease than others. These changes were also not progressive. For example, a plateau could be observed in the phylum *Proteobacteria* during the first 7 days of biofilm accumulation before it began to decrease [14].

Common oral hygiene practices in gingivitis would result in minimal changes at the taxonomic level. Brushing teeth with sodium fluoride toothpaste produced no change in the diversity of the microbiome in either the sub- or the supragingival biofilms. At the supragingival level, some significant changes could be observed in a few genera [23]. In fact, the anticaries effect of this compound is more likely to be due to other factors, such as increased pH or increased remineralization of enamel, rather than antibacterial activity [46].

When other ingredients, such as stannous fluoride or triclosan/copolymer, were used, changes in the diversity of the subgingival microbiome could be observed. These changes were only significant in the case of triclosan/copolymer [12], confirming its antibacterial activity [47]. In the case of stannous fluoride toothpaste, only two species showed a significant reduction (*P. endodontalis* and *T. forsythia*) [15]. It seems that toothbrushing during gingivitis would produce no changes or minimal changes in the total number of a few species, with no effect on the diversity or the relative abundance of most taxa, neither sub- nor supragingivally.

On the other hand, although it was not the objective of this review, it could be observed that PMPR plus toothbrushing could achieve a significant change in the subgingival diversity [13], with a positive impact on different genera [13,14].

### 3.4. Use of Antiseptic Mouth Rinses

CHX is known to be a potent broad-spectrum antimicrobial agent [48]. In the present review, CHX was found to have a marked subgingival effect on both the diversity and composition of the microbiome, affecting six phyla: *Fusobacteriota*, *Firmicutes*, *Bacteroidota*, *Actinobacteriota*, *Proteobacteria*, and *Candidatus Saccharibacteria* [21]. A significant decrease of 25 gingivitis-related species and an enrichment of four species, such as *Kingella oralis* or *Streptococcus* sp. HOT_423, associated with periodontal health [11,49], was also observed in only 14 days.

CPC mouth rinses were evaluated in supragingival biofilm. One study found a significant impact in terms of diversity and biofilm structure as early as day 11 [23], while another study only observed differences in beta diversity [17]. It should be noted that in the latter study, patients only used mouth rinse without toothbrushing. However, seven phyla were affected in both studies: *Fusobacteriota*, *Firmicutes*, *Bacteroidota*, *Actinobacteriota*, *Proteobacteria*, *Patescibacteria* group, and *Campylobacterota*, with an increase in the genus *Rothia* and *Streptococcus*, among others, and a reduction of disease-related genera, such as *Porphyromonas*, *Prevotella*, and *Tannerella*.

On the other hand, CPC plus essential oils only showed an effect on beta diversity, with contradictory results at the species level, because an increase in the relative abundance of *Fusobacterium nucleatum*, *T. forsythia*, and *Treponema socranskii* was observed [25]. The significance of this fact is difficult to interpret because the authors did not report the concentration of CPC used. The use of a NAC-based mouth rinse was not able to induce changes in the subgingival microbiome at any level [21].

### 3.5. Limitations and Future Considerations

The main limitation of the current work is methodological since we compared the results of studies using different taxonomic assignment methods (OTUs and ASVs) [50]. In addition, different sources of bias can be identified across the studies that could explain some of the discrepancies observed, such as the variable region amplified and the DNA amplification protocol [51]. Another source of bias may be the statistical approach to the data since some studies used traditional methods to analyze the differential abundance of taxa, which can lead to different false positive rates [52,53].

Additionally, it should be noted that the studies varied considerably in their designs. Not all experimental studies presented the three phases (pre-induction, induction, and resolution), and not all these phases lasted equally long. The number of studies evaluating each antiseptic was very small, including the number of subjects in each study. Furthermore, depending on the country and sampling method (curettes or paper points), there could be differences in the relative abundance of certain taxa. These could be due to regional variability in the composition of the microbiota, as previously reported [54], and to the fact that paper points would preferentially retrieve bacteria from the outer layer of the biofilm, whereas curettes would favor biofilm attached to the tooth [55].

Finally, we recommend the establishment of standardized protocols, including combinations of hypervariable regions and an ASV assignment approach, to ensure comparable and reliable results. In addition, it would also be necessary to reach a consensus on the appropriate way to analyze this type of data. On the other hand, further RCTs and a larger number of subjects would be necessary to investigate the effect of oral hygiene products on the gingivitis-associated microbiome at the species level, especially with CPC, and in the long term.

## 4. Materials and Methods

### 4.1. Information Sources and Search Strategy

A systematic electronic search was conducted on the MEDLINE^®^/PubMed^®^ database using the following keywords: “gingivitis” together with “experimental gingivitis”, “clinical trial”, and “microbiome” with the Boolean Operator AND. No date filter was used, and the last query date was 31 December 2023.

Selected studies were included according to the following criteria.

#### 4.1.1. Inclusion Criteria

- Articles published in English;
- Clinical studies in humans, both clinical trials and experimental models;
- Systemically healthy adult individuals ≥ 18 years of age;
- Studies evaluating the oral microbiome in biofilm-induced gingivitis and/or assessing changes after toothbrushing with toothpaste and/or mouth rinses in this condition (they could also include professional mechanical plaque removal (PMPR) or subgingival instrumentation);
- Evaluation of the composition of the oral microbiome by metabarcoding (amplification and sequencing of 16S rRNA gene) or metagenomics (whole shotgun metagenomic sequencing).

#### 4.1.2. Exclusion Criteria

- Studies assessing the composition of the oral microbiota by culture-dependent, immunological, polymerase chain reaction (PCR), checkerboard DNA hybridization, or microarray techniques;
- Subjects with other periodontal conditions, such as periodontal health (except in experimental gingivitis studies), periodontitis, peri-implantitis, and peri-implant mucositis;
- Subjects with relevant systemic diseases.

### 4.2. Data Extraction

The following information was independently extracted from the included studies and doubled-checked by two reviewers (V.V., M.I.): first author, year of the publication, country, study design, interventions, sample size, population studied, time frame of the study, method of microbial analysis, type of sample, time points of analysis, outcome measures, and results. Any discrepancies between reviewers were resolved by consensus. A third reviewer acted as an arbitrator (D.H.). A summary of the extracted data for each study is provided in Tables S1 and S2.

All studies were approved by local ethics committees in accordance with the Declaration of Helsinki. Informed consent was obtained from patients in most studies, although this was not mentioned in three of them [12,16,20].

### 4.3. Taxonomic Data

Four main types of standardization of taxon data were performed: (1) the suffix -ota was added to phyla whose names had already been validated by the International Code of Nomenclature for Prokaryotes [56]; (2) the names of the phyla *Bacillota* and *Pseudomonarota* were standardized across studies, and the older names were maintained as the most commonly used (*Firmicutes* and *Proteobacteria*, respectively) [57]; (3) phyla *Candidatus* were consulted on the NCBI Taxonomy website (https://www.ncbi.nlm.nih.gov/taxonomy, accessed on 30 March 2024), and their equivalents appear in the text; and (4) taxa named according to their 16S rRNA reference sequence were checked for taxonomic identity in the eHOMD 16S rRNA Reference Sequence Tree Version 15.23 database (https://www.homd.org/ftp//phylogenetic_trees/refseq/current/eHOMD_16S_rRNA_RefSeq.svg, accessed on 15 April 2024).

## 5. Conclusions

In biofilm-induced gingivitis, an increase in abundance and a gain of species could be observed with respect to periodontal health in both the sub- and the supragingival biofilms, leading to greater diversity. These result in a disturbance of the microbiome that is difficult to reverse with conventional oral hygiene practices, such as toothbrushing with fluoride toothpaste. Specific antiseptic agents, especially if delivered as mouth rinses, may have an impact on the supra- and subgingival microbiome in gingivitis.

## Figures and Tables

**Table 1:** Dynamics of species in the phyla *Bacteroidota*, *Fusobacteroidota*, *Spirochaetota*, and *Patescibacteria* groups at the end of the gingivitis induction phase, according to subgingival or supragingival samples [12,14,15,16,21,22].

Phylum	Genus	Subgingival		Supragingival	
		Increase	Decrease	Increase	Decrease
*Bacteroidota*	*Prevotella*	*Prevotella shahii*, *Prevotella loescheii*, *Prevotella saccharolytica*, *Prevotella micans*, *Prevotella maculosa*, *Prevotella oulorum*, *Prevotella marshii*, *Prevotella nigrescens*		*P. loescheii*, *P. saccharolytica*, *P. micans*, *P. maculosa*, *P. oulorum*, *Prevotella melaninogenica*, *Prevotella intermedia*	
	*Porphyromonas*	*Porphyromonas catoniae*, *Porphyromonas endodontalis*		*P. catoniae*	
	*Tannerella*	*Tannerella* sp. HOT_286		*Tannerella* sp. HOT_286	
	*Alloprevotella*	*Alloprevotella tannerae*, *Alloprevotella rava*			
	*Bergeyella*	uncultured *Bergeyella* sp.		uncultured *Bergeyella* sp.	
	*Capnocytophaga*		*Capnocytophaga gingivalis*, *Capnocytophaga leadbetteri*	*Capnocytophaga granulosa*, *Capnocytophaga* sp. HOT_B29	*Capnocytophaga sputigena*, *C. gingivalis*
*Fusobacteriota*	*Fusobacterium*	*Fusobacterium nucleatum* subsp. *vincentii*, *Fusobacterium nucleatum* subsp. *animalis*, *Fusobacterium nucleatum* subsp. *polymorphum*		*F. nucleatum* subsp. *polymorphum*	
	*Leptotrichia*	*Leptotrichia buccalis*, *Leptotrichia* sp. HOT_392		*L. buccalis*, *Leptotrichia* sp. HOT_212, *Leptotrichia* sp. HOT_223, *Leptotrichia* sp. HOT_225, *Leptotrichia* sp. HOT_417, *Leptotrichia hofstadii, Leptotrichia wadei, Leptotrichia shahii*	
*Spirochaetota*	*Treponema*	*Treponema medium*, *Treponema socranskii*, *Treponema maltophilum*, *Treponema vincentii*		*T. vincentii*	*T. socranskii*
*Patescibacteria* group ^a^	*Gracilibacteria* ^b^			*Gracilibacteria bacterium*	
	*Absconditabacteria*_(SR1)_[G-1] ^c^	*Absconditabacteria*_(SR1)_[G-1] sp. HOT_345		*Absconditabacteria*_(SR1)_[G-1] bacterium	
	*Saccharibacteria*_(TM7)_[G-1] ^d^	*Saccharibacteria*_(TM7)_[G-1] *bacterium*_HOT_346, *Saccharibacteria*_(TM7)_[G-1] *bacterium*_HOT_349		*Saccharibacteria*_(TM7)_[G-1] *bacterium*_HOT_347, *Saccharibacteria*_(TM7)_[G-1] *bacterium*_HOT_348, *Saccharibacteria*_(TM7)_[G-1] *bacterium*_HOT_349	

^a^: major bacterial phylogenetic group that includes various phyla; ^b^: *Candidatus Gracilibacteria* phyl., formerly known as GN02; ^c^: *Candidatus Absconditabacteria* phyl., formerly known as SR1; ^d^: *Candidatus Saccharibacteria* phyl., formerly known as TM7.

**Table 2:** Changes in different genera of the phylum *Firmicutes* at the end of the gingivitis induction phase, according to various studies and oral ecosystems.

Genus	Subgingival					Supragingival				
	Schincaglia et al., (2017) [12]	Al-Kamel et al., (2018) [21]	Bamashmou et al., (2021) [13]	Hall et al., (2023) [14]	Nowicki et al., (2018) [18]	Huang et al., (2014) [16]	Teng et al., (2016) [17]	Belstrøm et al., (2018) [20]	Huang et al., (2021) [19]	Hall et al., (2023) [14]
*Streptococcus* ^a^		↓	↓	↓	↓	↓	↓	↓	↓	↓
*Granulicatella* ^a^				↓				↓		↓
*Selenomonas* ^b^	↑		↑	↑		↑			↑	↓
*Dialister* ^b^				↑						NS
*Johnsonella* ^c^				↑		↑				↑
*Gemella* ^a^			↓	NS					NS	↑
*Parvimonas* ^d^				↑						NS
*Catonella* ^c^				↑		↑				↑
*Centipeda* ^b^				↑						↑
*Clostridia*_UCG-014 ^c^				↑						↑
*Peptostreptococcaceae*_[G-7] and [G-9] ^c^				↑		↑				NS
*Clostridia*_vadinBB60_group ^c^				NS						↓
*Peptococcus* ^c^				↑		↑			↑	NS

^a^: class *Bacilli*; ^b^: class *Negativicutes*; ^c^: class *Clostridia*; ^d^: traditionally classified in the class *Clostridia* but now considered to belong to the class *Tissierellia* [27]; NS: not statistically significant differences between the beginning and the end of the induction phase of gingivitis. Arrows indicate the direction of the change: increase (↑) or decrease (↓).

**Table 3:** Dynamics of species in the phyla *Firmicutes*, *Proteobacteria*, and *Actinobacteriota* at the end of the gingivitis induction phase, according to subgingival or supragingival samples [12,14,16,21,22].

Phylum	Genus	Subgingival		Supragingival	
		Increase	Decrease	Increase	Decrease
*Firmicutes*	*Streptococcus*		*Streptococcus mitis*, *Streptococcus sanguinis*, *Streptococcus gordonii*, *Streptococcus* sp. HOT_423, *Streptococcus* sp. HOT_064, *Streptococcus australis*	*Streptococcus cristatus*, *Streptococcus anginosus*	*S. mitis*, *S. sanguinis*
	*Peptostreptococcaceae*_[G-9]	[*Eubacterium*] *brachy* ^a^			
	*Clostridiales*	*Clostridiales bacterium*		*C. bacterium*	
	*Oribacterium*	*Oribacterium parvum*			
	*Selenomonas*	*Selenomonas sputigena*, *Selenomonas flueggei*, *Selenomonas infelix*, *Selenomonas* sp. HOT_G51		*S. sputigena*, *Selenomonas dianae*, *S. infelix*, *Selenomonas noxia*	
	*Peptostreptococcus*			*Peptostreptococcus stomatis*	
	*Peptococcus*			*Peptococcus* sp. HOT_167	
	*Johnsonella*			*Johnsonella ignava*	
	*Gemella*			*Gemella morbillorum*	
	*Dialister*			*Dialister invisus*	
	*Catonella*			*Catonella morbi*	
	*Granulicatella*			*Granulicatella elegans*	
	*Solobacterium*	*Solobacterium moorei*			
	*Lachnospiraceae*_[G-3]	*Lachnospiraceae*_[G-3] sp. HOT_100			
	*Mitsuokella*	*Mitsuokella* sp. HOT_521			
	*Staphylococcus*		*Staphylococcus hominis*, *Staphylococcus epidermidis*		
*Proteobacteria*	*Neisseria*		*Neisseria bacilliformis*	*Neisseria flavescens*	*N. bacilliformis*, *Neisseria elongata*
	*Haemophilus*		*Haemophilus parainfluenzae*, *Haemophilus haemolyticus*		*H. parainfluenzae*
	*Campylobacter*		*Campylobacter gracilis*	*Campylobacter showae*	
	*Cardiobacterium*	*Cardiobacterium valvarum*, *Cardiobacterium hominis*		*C. valvarum*	
	*Lautropia*				*Lautropia* sp. oral clone AP009
	*Kingella*		*Kingella oralis*		*Kingella oralis*
	*Aggregatibacter*			*Aggregatibacter* sp. HOT_513	
	*Pseudomonas*		*Pseudomonas otitidis*		
*Actinobacteriota*	*Propionibacterium*	*Propionibacterium propionicum*		*P. propionicum*	
	*Corynebacterium*		*Corynebacterium durum*		*C. durum*
	*Rothia*		*Rothia areia*, *Rothia dentocariosa*, *Rothia mucilaginosa*		*R. dentocariosa*, *R. aeria*
	*Actinomyces*			*Actinomyces dentalis*	*Actinomyces viscosus*, *Actinomyces naeslundii*
	*Actinobaculum*			*Actinobaculum* sp. HOT_848	
	*Brevibacterium*		*Brevibacterium casei*		

^a^: [ ] indicates that the name is awaiting appropriate action by the research community to be transferred to another genus.

**Table 4:** Microbiome dynamics at the species level following the use of chlorhexidine (CHX)-based mouth rinses at the subgingival level [21] and cetylpyridinium chloride (CPC) plus essential oils at the supragingival level [25].

Phylum	Subgingival (CHX)		Supragingival (CPC Plus Essential Oils)	
	Increase	Decrease	Increase	Decrease
*Bacteroidota*	*Capnocytophaga sputigena*, *Capnocytophaga gingivalis*	*Prevotella micans*, *Porphyromonas* sp. HOT_279, *Tannerella* sp. HOT_286	*Prevotella maculosa*, *Prevotella salivae*, *Prevotella* sp._HMT_292, *Prevotella* sp._HMT_376, *Prevotella tannerae*, *Tannerella forsythia*	*Bergeyella* sp. HMT_322, *Capnocytophaga leadbetteri*, *C. gingivalis*, *Porphyromonas pasteri*
*Fusobacteriota*		*Leptotrichia wadei*, *Leptotrichia* sp._HOT_225, *Leptotrichia* sp._HOT_392	*Fusobacterium nucleatum* subsp. *animalis*, *Fusobacterium nucleatum* subsp. *vincentii*	*Fusobacterium periodonticum*, *Fusobacterium nucleatum* subsp. *polymorphum*, *Fusobacterium* sp._HMT_370, *Leptotrichia hofstadii*, *Leptotrichia* sp._HMT_225
*Spirochaetota*			*Treponema parvum*, *Treponema lecithinolyticum*, *Treponema socranskii*, *Treponema* sp._HMT_231, *Treponema* sp._HMT_270	
*Candidatus Saccharibacteria*		*Saccharibacteria*_(TM7)_[G-1] *bacterium*_HOT_346, *Saccharibacteria*_(TM7)_[G-1] *bacterium*_HOT_349		
*Firmicutes*	*Streptococcus* sp. HOT_423	*Gemella haemolysans*, *Gemella morbillorum*, *Lachnoanaerobaculum umeaense*, *Lachnospiraceae*_[G-3] sp._HOT_100, *Mogibacterium diversum*, *Selenomonas infelix*, *Selenomonas noxia*, *Streptococcus cristatus*, *Streptococcus dentisani*, *Streptococcus sanguinis*, *Veillonella parvula* group	*Clostridiales*_[G-2] *bacterium*_HMT_085, *Dialister invisus*, *Mogibacterium timidum*, *Parvimonas* sp._HMT_110, *Peptococcus* sp._HMT_168, *Peptostreptococcaceae*_[G-4] *bacterium*_HMT_369, *Streptococcus constellatus*, *Streptococcus intermedius*, *Streptococcus* sp._HMT_066, *Veillonellaceae*_[G-1] *bacterium*_HMT_132	*S. dentisani*, *Streptococcus mitis*, *Streptococcus sinensis*
*Proteobacteria*	*Kingella oralis*	*Cardiobacterium hominis*, *Cardiobacterium valvarum*	*Campylobacter gracilis*, *Campylobacter* sp._HMT_044	*Aggregatibacter* sp._HMT_458, *Aggregatibacter* sp._HMT_513, *C. valvarum*, *Haemophilus parainfluenzae*, *Lautropia mirabilis*, *Neisseria flavescens*, *Neisseria subflava*, *Neisseria bacilliformis*, *Neisseria pharyngis*
*Actinobacteriota*		*Actinomyces johnsonii*, *Actinomyces naeslundii*, *Atopobium parvulum*, *Corynebacterium matruchotii*	*Actinomyces gerencseriae*, *Actinomyces naeslundii*, *Actinomyces* sp._HMT_169, *Actinomyces* sp._HMT_175, *Actinomyces* sp._HMT_525, *Rothia mucilaginosa*	*Actinomyces* sp._HMT_171, *Corynebacterium durum*, *Corynebacterium matruchotii*
*Synergistota*			*Fretibacterium* sp._HMT_359	

## Data Availability

No new data were created or analyzed in this study. Data sharing is not applicable to this article.

## Supplementary Materials

The following supporting information can be downloaded at https://www.mdpi.com/article/10.3390/antibiotics13060571/s1, Table S1: Design and characteristics of the included experimental gingivitis studies; Table S2: Design and characteristics of randomized controlled trials; Table S3: Methodological characteristics of the studies included in this review.
