# Supplementary material for: Supra- and Subgingival Microbiome in Gingivitis and Impact of Biofilm Control: A Comprehensive Review
Source: Antibiotics (Basel). 2024 Jun 20;13(6):571. doi: 10.3390/antibiotics13060571 (PMC11200379; doi:10.3390/antibiotics13060571)
Supplement: Supplementary file 1 [file antibiotics-13-00571-s001.zip › antibiotics-3041221-supplementary.pdf]

antibiotics

MDPI

Review

Supra- and Sub-Gingival Microbiome in Gingivitis and Impact Of Biofilm Control: A Comprehensive Review

Margarita Iniesta [1,2,]*, Viviane Vasconcelos [2], Mariano Sanz [1,2] and David Herrera [1,2]

[1] ETEP (Etiology and Therapy of Periodontal and Peri-implant Diseases) Research Group. Faculty of Dentistry, Complutense University of Madrid, Spain.
[2] Section of Graduate Periodontology, Department of Dental Clinic Specialties, Faculty of Dentistry, Complutense University of Madrid, Spain.
* Correspondence: margaini@ucm.es

Abstract: This comprehensive review was aimed (1) to characterize the sub- and supra-gingival microbiome in patients with biofilm-induced gingivitis (including experimental gingivitis), (2) to assess its stability and evolution over time, and (3) to assess the impact of biofilm control measures on this stability. An electronic search of the MEDLINE/PubMed® database until December 2023 was conducted. NCBI Taxonomy, eHOMD 16S rRNA Reference Sequence and Tree Version 15.23 databases were used to standardize taxonomic nomenclature. Out of 89 papers initially retrieved, 14 studies were finally included: 11 using experimental gingivitis as a model, and 3 randomized clinical trials evaluating the impact of biofilm control measures. Among them, 5 characterized the subgingival microbiome, 9 the supragingival microbiome, and 1 both the sub- and supra-gingival microbiome. In addition, 5 studies evaluated the effect of toothpastes and 4 studies evaluated the effect of mouth rinses. The diversity and structure of the microbiome differed significantly between patients with periodontal health and those with biofilm-induced gingivitis (including experimental gingivitis). Those differences were not reversed by conventional oral hygiene measures. Specific antiseptic agents, especially if delivered as mouth rinses, may have an impact on the supra- and sub-gingival microbiome in gingivitis.

Keywords: microbiome; biofilm control; gingivitis; toothpaste; mouth rinse; metabarcoding

Table S1. Design and characteristics of the included experimental gingivitis studies.

Author/Country	Pre-induction phase	Induction phase	Resolution phase	Groups	Inclusion criteria	Exclusion criteria	Outcomes	Main results
Fine et al. 2023/ Canada	**Day-21:** 0.76% MFP TP**; day-14:** using test or control products.	**Day 0:** abstaining from all oral hygiene practices.	**Day 21**: dental prophylaxis and brushing with test or control products was reinstituted; **day 42:** final plaque samples.	**Control group:** n=17 (NaF TP); **Test group**: n=16 (SnF2 stabilized with zinc phosphate TP).	Oral and periodontal health participants between 18 and 70 years of age; non-smokers.	Active or history of periodontitis or systemic conditions.	Alpha diversity<br>Relative abundance: phylum, genus, and species	**Alpha diversity:** increase in both groups at day 21 (NS). Test group lower diversity at 0 and day 42 (NS).<br>**Induction phase**<br>**Test group at day 0:** higher abundance of *Fusobacteriota*. Lower abundance of *Porphyromonas endodontalis* and *Treponema denticola*.<br>**Resolution phase**<br>**Test group at day 42:** lower abundance in the phyla *Bacteroidota* and *Spirochaetota*, in the genera *Treponema* and *Bacteroidales* G-2; and in the species *P. endodontalis* and *Tannerella forsythia*.
Hall et al. 2023/ USA	**Day-7:** scaling, dental cleaning and oral hygiene instructions.	**Day 0:** abstaining from all oral hygiene practices; **day 7 and day 14:** plaque samples; **day 21**: dental prophylaxis and normal hygiene practices.	**Day 28 and 35**: plaque samples.	**One group:** n=15	Systemically healthy, with no active or previous history of periodontal disease; chronic aphthous ulcers or tonsillitis; no more than four active or filled cavitated lesions.	Pregnant; past or current smokers; users of nonsteroidal anti-inflammatory or anti-microbial drugs, mouth rinses, or vitamin supplements within the previous three months.	Alpha diversity<br>Beta diversity<br>Relative abundance: phylum, genus, and species	**Alpha diversity:** increase at day 21 (NS). The change was bigger in subgingival samples.<br>**Beta diversity:** Within-subject dissimilarities were lower than between-subject.<br>**Induction phase**<br>10 phyla, 40 genera, and 43 species increased or decreased
Huang et al. 2021/ China	**Day -21, -14, -7:** rigorous oral-hygiene regimen (dental scaling and prophylaxis); NaF TP twice day, 3 min.	**Day 0, 1, 3, 7, 14, 28:** abstaining from all oral hygiene practices; **day 28:** final plaque samples.		**One group:** n=40	Healthy subjects aged 18 years or older who had a minimum of 18 natural teeth; ≥ 15 bleeding sites.	Use of antibiotic, anti-inflammatory or anticoagulant therapy within 30 days prior to examination; pregnancy or lactation; diabetes; a history of hepatitis or blood disorders; presence of orthodontic appliances or removable partial dentures; significant oral pathology, such as advanced periodontal disease, hard or tissue tumors, or other conditions.	Beta diversity<br>Relative abundance: genus	**Beta diversity:** changes observed from 1-3 days.<br>**Induction phase:** changes observed<br>At day 0: 14 genera<br>At day 1: 4 genera<br>At day 3: 11 genera<br>At day 7: 11 genera<br>At day 14: 16 genera<br>At day 28: 16 genera
Bamashmous et al. 2021/ USA	**Day-14:** prophylaxis and normal hygiene.	**Day 0, 7, 14, 21:** intraoral stents to prevent accidental brushing of the experimental sites.	**Day 21 to day 35:** dental prophylaxis and normal hygiene (electric toothbrush [Philips Sonicare	**One group:** n=21	Healthy subjects aged 18-35 years; no clinical signs of gingival inflammation at >90% of sites at time of screening	Medical condition which requires premedication; history of periodontal disease; history of systemic inflammatory or immune conditions; use of antibiotics or anti-inflammatory	Alpha diversity<br>Beta diversity<br>Relative abundance: phylum, genus, and species.	**Alpha diversity:** increase at day 21 corresponding to increased gingivitis severity. Different points in time and indices.<br>**Beta diversity:** changes observed from day 4. The slow group showed a

			Electric Toothbrush], Colgate® Cavity Protection TP and dental floss (Oral B Glide).		(GI=0); no signs of "periodontal disease".	drugs within 30 days of enrolment; pregnant or breastfeeding; orthodontic treatment; untreated carious lesion; use of tobacco; removable partial dentures; history of allergy to common dentifrice ingredients; immune compromised individuals.	Three different response groups: high, low, and slow responders.	separation from the high and low groups.<br>**Induction phase**<br>Firmicutes and *Actinobacteriota* decreased, and *Bacteroidota* increased.<br>-Variations among the groups: *Tannerella* increased late in gingivitis for both low and slow groups but was lower than in the high response group. *Prevotella* increased late in the low and high group but was lower than in the slow group. Higher abundance of *Streptococcus sanguinis* and *Streptococcus oralis* in the slow group.<br>**Resolution phase**<br>Higher abundance of *S. sanguinis* and *S. oralis* in the slow group and a tendency to pre-induction levels.
Al- Kamel et al. 2019/ Yemen		**Day 0:** abstaining from all oral hygiene practices and allocated into NAC, CHX, or Control MR group.	**Day 21:** 20 subjects allocated into NAC or CHX MR; **Day 35:** final samples.	**Control group:** n=10 (base formula); **Test group**: n=10 (1.25% NAC MW, 15 ml) and n=10 (0.2% CHX MR, 15 ml).	BOP ≤ 10% of the sites; no evidence of detectable interdental CAL at ≥ 2 non-adjacent teeth, or nondetectable buccal CAL ≥ 3 mm with probing ≥ 3 mm at ≥ 2 teeth; 18 to 28 years old; at least 6 teeth per quadrant; non-smokers.	Qat chewing; pregnancy or breastfeeding; systemic diseases known to modify gingival health; no history of use of antibiotics, antifungal, corticosteroids or contraceptives within last 3 months; absence of major retentive factors such as orthodontic and prosthodontic appliances.	Alpha diversity<br>Beta diversity<br>Relative Abundance: phylum, genus, and species	**Alpha diversity**: decrease in CHX group, with NS change in placebo and NAC groups.<br>**Beta diversity:** differences in composition between day 0 and 21. NAC group clustered with placebo group.<br>**Induction phase**<br>-Placebo group: increase of 13 species.<br>-NAC group: increase of 16 species.<br>-Increased species in both groups: TM7 G1 sp. oral<br>taxon 346, *Cardiobacterium valvarum*, *Cardiobacterium hominis*, and *Porphyromonas*<br>*catoniae*.<br>-CHX *group*: decrease of 35 species, and increase of *Granulicatella adiacens*.<br>**Resolution phase**<br>-NAC group: NS changes<br>-CHX group:<br>Phylum: decrease of TM7, SR1, and *Actinobacteriota*.<br>Genus: decrease of 14 genera, and increase of *Capnocytophaga*.<br>Species: decrease of 25 species, and increase of 4 species.
Nowicki et al. 2018/ USA	**Day-14:** dental prophylaxis and oral hygiene instructions.	**Day 0,3,21:** intraoral stents to preventing accidental brushing		**One group:** n= 10	Healthy subjects; age ≥18 years; a minimum 20 natural teeth; baseline MGI ≤ 1.	Medical conditions with prophylactic antibiotic; antibiotic, anti-inflammatory, or anticoagulant medication within 1 month prior to the study;	Alpha diversity<br>Beta diversity<br>Relative Abundance: genus	**Alpha** diversity**:** NS changes between groups. In contrast, high and medium bleeding subjects showed significant differences compared to the low bleeding group.

		of the experimental sites.				smokers; pregnancy or breastfeeding; oral tissue pathology (excluding gingivitis); chronic periodontitis; underlying genetic or immunologic condition that might influence the study.		**Beta diversity:** differences between subjects scored the highest and the lowest gingival index.<br>**Induction phase**<br>-Non-bleeding sites: increase of *Streptococcus*, *Neisseria*, and *Lautropia*.<br>-Gingivitis sites: increase of *Oribacterium*, *Leptotrichia*, *Tannerella*,,and *Lachnoanaerobaculum*.
Belstrøm et al. 2018/ Denmark		**Day 0,4,7,10**: abstaining from all oral hygiene practices.	**Day 10 to day 24**: dental prophylaxis, normal hygiene and final sample.	**One group:** n= 29	Age ≥18	Caries, gingivitis, or periodontitis (presence of 1 site with probing pocket depth ≥4 mm + bleeding on probing); hyposalivation; smokers; systemic diseases and current use of any medication; use of antibiotics within the past 3 months; professional dental cleaning within the past 3 months.	Beta diversity<br>Relative abundance: genus and species	**Beta diversity:** changes observed from day 4.<br>**Induction phase**<br>-Increase of *Leptotrichia*, and *Corynebacterium*.<br>-Decrease of *Streptococcus, Prevotella, Rothia, Haemophilus,* and *Granulicatella*.<br>-75 species increased or decreased.
Teng et al. 2016/ China	**Day-21:** dental prophylaxis and oral hygiene instructions. No plaque samples at this phase.	**Day 0, 7, 14, 21:** abstaining from all oral hygiene practices and allocation to a MR group for 30 s.		**Control group:** n=50 (20 mL of water); **Test group:** n=41 (20 ml CPC).	Healthy subjects; age ≥18 years; a minimum of 12 natural anterior teeth; ≤ 5 bleeding sites at day -21.	Periodontitis (four teeth with ≥5 mm pockets in two quadrants); purulent exudates, generalized mobility, and/or severe recession; use of antibiotics any time during the study or premedication; pregnancy or breastfeeding; orthodontic appliances; atypical discoloration or pigmentation in the gingival tissue; any diseases or conditions that could be expected to interfere with the subject safely completing the study.	Alpha diversity<br>Beta diversity<br>Relative Abundance: genus	**Alpha diversity:** increase in the control group.<br>**Beta diversity:** differences in composition between day 0 and 21 in both groups. Values of CPC group lower than control group.<br>**Induction phase**<br>-Control group: increase of *Porphyromonas*, *Corynebacterium*, *Abiotrophia* and TM7.<br>-Test group: decrease of 17 genera, and increase of *Haemophilu*s, *Lautropia*, *Neisseria*, *Capnocytophaga,* and *Propionibacterium.*
Schincaglia et al. 2017/ USA	**Day-14:** dental scaling and prophylaxis, oral hygiene instructions.	**day 0, 7, 14, 21:** intraoral stents to preventing accidental brushing of the experimental sites.	**day 21 to 42**: Colgate Total® TP and final sampling.	**One group:** n= 15	Age ≥ 21 years; absence of active infection; absence of radiographic bone loss; probing depth ≤4mm.	Antibiotic use within 1 month; systemic diseases requiring the use of anti-inflammatory, antibiotics, anticoagulants; any medication initiated <3 months prior; caries, periodontal diseases; smokers; any medications known to affect periodontal health; pregnancy; use of antimicrobial rinses or irrigating device; acute dental/oral condition requiring treatment.	Alpha diversity<br>Beta diversity<br>Relative Abundance: genus and species	**Alpha diversity:** increase at day 21, and decrease at day 42.<br>**Beta diversity:** differences in composition between day 0 and 21.<br>**Induction phase**<br>Increase of genus *Selenomonas*, and *Selenomonas flueggei* HOT 125, and *Prevotella nigrescens* HOT 693.<br>Decrease of genera *Rothia* and *Stenotrophomonas*.<br>**Resolution phase**<br>Taxa tend to initial levels.

Huang et al. 2014/ China	**Day -21, -14, -7:** oral hygiene instructions.	**Day 0 to day 21:** abstaining from all oral hygiene practices.		**Main group:** n= 50	Healthy subjects; age ≥ 18 years; a minimum of 18 natural teeth; ≥ 15 bleeding sites.	Use of antibiotic, anti-inflammatory or anticoagulant therapy within 30 days before; pregnancy or breastfeeding; diabetes; a history of hepatitis or blood disorders; orthodontic appliances; removable partial dentures; advanced periodontal disease, hard or tissue tumors.	Beta diversity<br>Relative abundance: phylum, genus, and species	**Beta diversity:** differences in composition between day 0 versus -21 and 21.<br>**Induction phase**<br>-Phylum: increase of *Fusobacteriota, Bacteroidota,* and TM7; and decrease of *Actinobacteriota* and *Firmicutes*.<br>-Genus: increase of 22 genera; and decrease of *Streptococcus*, *Rothia*, *Actinomyces*, *Haemophilus,* and *Lautropia*.<br>-Species: 78 species increased or decreased.
Kistler et al. 2013/ United Kingdom		**Day 0 to day-14:** abstaining from all oral hygiene practices in the mandible.		**Main group**: n=20	Health subjects; a minimum 20 teeth; no history of antibiotic use for at least three months prior to the study.	Pregnancy; current smokers or individuals who quit smoking within the previous five years.	Alpha diversity<br>Beta diversity<br>Relative abundance: phylum and species	**Alpha diversity**: increase at day 14.<br>**Beta diversity**: differences in composition between day 0 and 14.<br>**Induction phase**<br>-Phylum: increase of Bacteroidota and decrease of *Actinobacteriota*.<br>-Species: increase of 21 species; and decrease of 10 species.

MFP: sodium monofluorophosphate; TP: toothpaste; NaF: sodium fluoride; GI: gingival index; NAC: N-acetyl cysteine; CHX: chlorhexidine; MR: mouth rinse; MGI: Mazza gingival index; BOP: bleeding on probing; CAL: clinical attachment loss; CPC: cetylpyridinium chloride; NS: not significant.

Table S2. Design and characteristics of randomized controlled trials.

Author/Country	Design	n	Control group	Test group	Inclusion criteria	Exclusion criteria	Outcomes	Main results
Newman et al. 2022/ USA	**Day 0**: dental prophylaxis and allocated into groups; **day 42 and 84**: sample collection.	80	n= 41 (water rinse)	n= 39 (CPC + Essential Oil)	Healthy subjects: age 18-60 years; a minimum 20 teeth, with ≥10 bleeding sites at baseline.	Periodontal disease with purulent exudate, mobility and/or recession; current treatment for periodontal disease; bleeding disorder or use of a blood thinner; orthodontic treatment; diabetes; pregnancy; antibiotic therapy within 3 months; any diseases or conditions that may interfere.	Alpha diversity<br>Beta diversity<br>Relative abundance: species	**Alpha diversity**: increase in both groups at day 84 (NS).<br>**Beta diversity**: differences in composition between day 0 and 84 in test group.<br>**Test group**<br>Increase of 40 species; and decrease of 33 species (among these: *Corynebacterium matruchotii*, *Corynebacterium durum*, and species of *Actinomyces, Fusobacterium, Leptotrichia, Capnocytophaga, Neisseria, Streptococcus, Aggregatibacter, Porphyromonas, Lautropia,* and *Terrahaemophilus aromaticivorans*).
Keijser et al. 2018/ Netherlands	**Day 0:** oral hygiene instructions with NaF TP twice a day (HEMA®) and allocated into groups; **day 28 and 42**: sample collection.	153	n= 53 (no chewing gum) plus n=50 (use of gum base, five times daily during 10 min).	n=50 (maltitol gum, five times daily during 10 min).	Healthy subjects; age 18–45 years; non-smokers; ≥ 20 natural teeth (five per quadrant).	Dental caries and periodontitis; orthodontic and prosthodontic appliances; night guards; oral and/or peri-oral piercings; oral lesions; antibiotics in the preceding 2 months; pregnancy and breast feeding.	Beta diversity<br>Relative abundance: phylum, genus, and species	**Beta diversity:** differences in composition between day 0 and 28.<br>**Control group** (NS changes)<br>-Phylum: increase of *Actinobacteriota*; and decrease of *Firmicutes*, *Fusobacteriota,* and *Bacteroidota*.<br>-Genus: increase of *Actinomyces* and *Corynebacterium*; and decrease of *Leptotrichia*, *Fusobacterium*, and *Capnocytophaga*.<br>-Species: No table or figure shows the control group change.
Huang et al. 2016/ China	**Day 0:** allocation into groups; **day 3, 11 and 27:** sample collection.	91	n=44 (brush-alone group, twice-daily with a Cross Action manual brush [Crest®] plus a 0.243% NaF TP [Crest®]).	n=47 (brush-plus-rinse group, using a Cross Action manual brush [Crest®] and a 0.321% NaF TP (Crest®) plus 0.0747% CPC MR [Crest®] 20ml/30 sec after brushing).	Healthy subjects; age ≥18 years; a minimum 18 natural teeth; ≥ 10 bleeding sites (MGI 1.0–2.5 at baseline).	Severe periodontal disease (≥ 4 teeth with ≥ 5 mm probing depth in two quadrants, purulent exudates, mobility, and/or severe recession); any condition that requires antibiotic premedication; pregnancy and breastfeeding; orthodontic treatment; atypical discoloration or pigmentation in the gingival tissue; any diseases or	Alpha diversity<br>Beta diversity<br>Relative abundance: genus	**Alpha diversity:** decrease in test group between day 11 and 27.<br>**Beta diversity:** differences in composition between groups.<br>**Control group**<br>Decrease of Actinobaculum, TM7, and *Leptotrichia*; and increase of *Actynomices*.<br>**Test group**

						conditions that could be expected to interfere with the subject safely completing the study.		Decrease of 13 genera (including *Actinobaculum,* TM7, and *Leptotrichia*); and increase of *Lautropia*, *Rothia,* and *Streptococcus*.

CPC: cetylpyridinium chloride; NaF: sodium fluoride; TP: toothpaste; MR: mouth rinse; MGI: Mazza gingival index; NS: not significant.

Table S3. Methodological characteristics of the studies included in this review.

Author/Country	Type of sample	Sampling method	Amplicon	Feature	Database
**Experimental gingivitis**					
Fine et al. 2023/ Canada	Subgingival	Paper point	16S rRNA gene (V3-V4)	ASV	HOMD
Hall et al. 2023/ USA	Subgingival and supragingival	Curette	16S rRNA gene (V4-V5)	ASV	HOMD
Huang et al. 2021/ China	Supragingival	Curette	16S rRNA gene (V1-V3)	OTU	Oral CORE
Bamashmous et al. 2021/ USA	Subgingival	Paper point	16S rRNA gene (V3-V4)	ASV	HOMD
Al- Kamel et al. 2019/ Yemen	Subgingival	Paper point	16S rRNA gene (V1-V3)	OTU	HOMD
Norwicki et al. 2018/ USA	Subgingival	Paper strip	16S rRNA gene (V4-V5)	OTU	HOMD
Belstrøm et al. 2017/ Denmark	Supragingival	Periodontal probe	16S rRNA gene (V3-V4)	OTU	HOMD, GreenGenes, GenBank and Ribosomal Database Project
Teng et al. 2016/ China	Supragingival	Curette	16S rRNA gene (V1-V3)	OTU	Oral CORE
Schincaglia et al. 2016/ USA	Subgingival	Curette	16S rRNA gene (V1-V2)	OTU	HOMD
Huang et al. 2014/ China	Supragingival	Curette	16S rRNA gene (V1-V3)	OTU	Oral CORE
Kistler et al. 2013/ United Kingdom	Supragingival	Curette	16S rRNA gene (V1-V3)	OTU	HOMD
**Randomized controlled trial**					
Newman et al. 2022/ USA	Supragingival	Curette	16S rRNA gene (V1-V3)	OTU	HOMD
Keijser et al. 2018/ Netherlands	Supragingival	Teflon spatula	16S rRNA gene (V4)	OTU	HOMD
Huang et al. 2016/ China	Supragingival	Curette	16S rRNA gene (V1-V3)	OTU	Oral CORE

ASV: amplicon sequence variants; OUT: operational taxonomic units; HOMD: Human Oral Microbiome Database.
